# Torticollis

**Published:** 1891-03-21

**Authors:** 


### SURGERY.

### TORTICOLLIS.

Torticollis may be divided for practical purposes into three classes—First, the so-called "false torticollis," where the condition is due to osseous deformity, cervical caries for example, and admits of no remedial measures.

Second, fixed wry-neck, which is generally congenital, but may arise in adult life from injury to muscles, producing inflammation with secondary cicatricial contraction. Here the treatment is simple and effectual, consisting in division of the contracted tissues, and keeping the head fixed in the normal position for 10 or 15 days.

Third, spasmodic wry-neck. This, the most distressing as it is the most rebellious form of torticollis, calls for more particular comment. The pathology of this affection is unknown. Cowen, in his book on nervous diseases, although pointing out that the muscular grouping in different cases of this kind suggests that in some cases there is a cerebral and in others a spinal origin, yet sums up by saying, "it seems doubtful whether we have at present sufficient evidence to justify us in coming to any conclusion regarding the seat of the disease." This, then, being the state of our knowledge, we are driven to a consideration of the results obtained by different observers as a guide to the best method of treat ment.

The various means employed may be arranged under two heads—sedative, and what we call paralytic, that is to say measures which have for their object the temporary or permanent paralysis of the nerve supplying the affected muscles.

(I.) Under the head of sedative treatment come the various drugs that have been used, generally in large doses, viz., opium or morphia, chloral, succus conii, bromide of potassium, Indian hemp, and gelsemium, as lately recommended by C. B. Williams, in toxic doses.

Only one of these—morphia hypodermically administered—seems to be as a rule at all effectual, but in this case the serious danger of inducing a morphia habit must never be lost sight of, for the drug has to be continued for many months, as a return of the spasms is very likely to occur, or the patient fears it if the injections are left off.

In the same class we must put galvanism of the affected muscles, this having a sedative influence. Moriz Benedikt who regards the disease as a reflex neurosis, reports three cases treated with an alternating current—in two he also used injections of a 2 per cent. sol. of carbolic acid and these were cured; the other was markedly benefited.

Gowers however, has not found this method of treatment successful.

(II.) The paralytic treatment consists in stretching or excising a portion of the spinal accessory nerve of the affected side. This nerve furnishes nearly the entire supply of the sterno-mastoid, although a few filaments are also sent to it from the second cervical nerve, but supplies only the upper part of the trapezius.

The evidence in regard to the efficacy of nerve-stretching and neurectomy is very conflicting. Gowers says that most of the cases so treated are either aggravated or if relieved for the time relapse again.

Page reports a case coming on after an injury to the cervical spine which had resisted all forms of treatment and where nerve-stretching entirely relieved the condition, only a slight rigidity of the muscles remaining.

Renton records a case due to cold where the spinal accessory was stretched with temporary complete relief, the spasms recurring, but only slightly.

Moriz Benedikt also published three cases so treated with excellent results. Richardson and Warren recommended division of the nerve, and it seems generally safer to excise a portion, say an inch, to prevent restoration of its function.

Within the last month Mr. Southam has published three more cases in which he has successfully carried out this treatment. He makes an incision along the anterior border of the sterno-mastoid opposite the angle of the jaw, and excises about one-third inch of the nerve just as it is passing behind the muscle. After the operation an apparatus is worn consisting of a poroplastic cap and a waist band between which is stretched an elastic band in a direction parallel with the line of action of the unaffected sterno-mastoid. This has to be worn for some months perhaps, and passive rotatory movements done daily. In two of the cases eighteen and six months respectively have elapsed since the operation, and the result is excellent; the third case has only been under observation for a month, but is so far satisfactory. Mr. Southam considers cases of the tonic variety of spasmodic wry-neck, and especially those where the sterno-mastoid is the only or main muscle affected, to be most suitable for this treatment, although he has had a good result in a case of the clonic variety, and where many muscles were implicated. It seems, therefore, well worthy of trial in any case of the spasmodic group which has resisted other less severe treatment. No mention has been made of "hysterical torticollis," as its treatment must be entirely on the lines of the general neurosis. Age is a valuable aid to diagnosis true torticollis very seldom appearing before 30 years of age.

